# Potentials of on-line repositioning based on implanted fiducial markers and electronic portal imaging in prostate cancer radiotherapy

**DOI:** 10.1186/1748-717X-4-13

**Published:** 2009-04-27

**Authors:** Reinhold Graf, Peter Wust, Volker Budach, Dirk Boehmer

**Affiliations:** 1 Charité Universitätsmedizin Berlin, Department of Radiotherapy, Campus Virchow-Klinikum, Augustenburger Platz 1, 13353 Berlin, Germany

## Abstract

**Background:**

To evaluate the benefit of an on-line correction protocol based on implanted markers and weekly portal imaging in external beam radiotherapy of prostate cancer. To compare the use of bony anatomy versus implanted markers for calculation of setup-error plus/minus prostate movement. To estimate the error reduction (and the corresponding margin reduction) by reducing the total error to 3 mm once a week, three times per week or every treatment day.

**Methods:**

23 patients had three to five, 2.5 mm Ø spherical gold markers transrectally inserted into the prostate before radiotherapy. Verification and correction of treatment position by analysis of orthogonal portal images was performed on a weekly basis. We registered with respect to the bony contours (setup error) and to the marker position (prostate motion) and determined the total error. The systematic and random errors are specified. Positioning correction was applied with a threshold of 5 mm displacement.

**Results:**

The systematic error (1 standard deviation [SD]) in left-right (LR), superior-inferior (SI) and anterior-posterior (AP) direction contributes for the setup 1.6 mm, 2.1 mm and 2.4 mm and for prostate motion 1.1 mm, 1.9 mm and 2.3 mm. The random error (1 SD) in LR, SI and AP direction amounts for the setup 2.3 mm, 2.7 mm and 2.7 mm and for motion 1.4 mm, 2.3 mm and 2.7 mm. The resulting total error suggests margins of 7.0 mm (LR), 9.5 mm (SI) and 9.5 mm (AP) between clinical target volume (CTV) and planning target volume (PTV). After correction once a week the margins were lowered to 6.7, 8.2 and 8.7 mm and furthermore down to 4.9, 5.1 and 4.8 mm after correcting every treatment day.

**Conclusion:**

Prostate movement relative to adjacent bony anatomy is significant and contributes substantially to the target position variability. Performing on-line setup correction using implanted radioopaque markers and megavoltage radiography results in reduced treatment margins depending on the online imaging protocol (once a week or more frequently).

## Background

There is evidence that dose-escalation in definitive radiotherapy of prostate cancer improves long-term PSA control [1]. One strategy to reduce late side effects is employment of gradually smaller radiation field sizes or planning target volumes PTV [2]. Tight margins will decrease the volume dose delivered to organs at risk, thus increasing the therapeutic ratio of **t**umor **c**ontrol **p**robability versus **n**ormal **t**issue **c**omplication **p**robability (TCP/NTCP). On the other hand, this ratio might decline if the clinical target volume is partially missed by any positioning error not compensated by the specified safety margins [3].

Retrospective evaluations [4,5] have suggested that anatomic variations (rectal distension, large rectum) during the planning CT in fact reduce the PSA control. A large (distended) rectum during planning can cause a systematic error, because it places the prostate more anterior, but this location might change from fraction to fraction. Another study did not confirm a correlation between rectal and/or bladder distension and errors of prostate position [6]. Nevertheless, we assume that image-guidance is crucial and improves the clinical outcome.

An assessment of patient position is based on skeletal landmarks imaged by electronic portal imaging devices (EPID). They are commonly used for the evaluation and correction of set-up deviations [7].

As documented in a number of studies [8,9], an interfractional displacement of the prostate itself can occur during radiation therapy fractions relative to the bony structures of the pelvis. The feasibility of implanting markers for localization of the prostate recently has been demonstrated [10,11] and allows to utilize EPIDs to quantify the displacement of the target [12,13]. With the improvement of online imaging quality, pretreatment localization and online protocols allowing positioning corrections without significant delay have gained feasibility [14].

From the comparison of verification protocols during radiotherapy it is known, that the treatment margins are institution specific. We performed a prospective study of patients treated with conformal radiotherapy for prostate cancer, analysing both internal organ motion and setup error with the objective to quantify the variability in prostate position. For displacements of bones and markers, statistical data including overall, systematic and random deviations were determined. From the uncorrected and corrected total errors, we calculated the necessary treatment margins to ensure sufficient target coverage in the majority of cases.

## Patients and methods

Verification and correction of treatment position by analysis of portal images and simulator control films were performed weekly for 23 patients with histologically confirmed prostate cancer treated from 1996 to 2000. The majority of patients were treated by a standard irradiation regimen in combination with regional hyperthermia in a phase II study as previously described [15]. Informed consent had been obtained from all patients.

Before treatment planning, three to five spherical gold (99.9% Au) markers with a diameter of 2.0 mm were inserted transrectally into the prostate of each patient using a modified biopsy needle under ultrasound guidance and local anaesthesia. Usually three markers were implanted, one into the apex, and two into the superior lateral parts of the prostate. Gold markers of this size can be visualized using megavoltage beam detector systems of the first generation. No complications occurred in association with the implantation process as reported elsewhere [10]. Note that the gold markers presently applied with kV X-ray tracking systems are < 1 mm in diameter and the implantation procedure is easier and more feasible.

Each patient underwent a computerized tomography scan (CT) (Siemens™, Erlangen, Germany) for treatment planning in treatment position from 2 cm below the ischial tuberosities to the L4/5 interspace obtaining volumetric data at 5 mm slice thickness and at a 5 mm couch translation. In our study, the patients were instructed to fill the bladder, but no effort was made to control the rectal volume. However, the CT scans were repeated if excessive filling of the rectum had been noticed. Patients were stabilized in supine position with conventional head, knee and feet support and no rigid immobilization device was used. Images were transferred to a workstation (Helax™) for anatomic segmentation of targets and organs at risk and conformal dosimetric planning. The PTV was defined by a three-dimensional expansion of the CTV by 8 mm at the prostate-rectum interface and 10 mm in all other directions. External beam radiotherapy was performed by a linear accelerator (Siemens™ Mevatron KD, Erlangen, Germany) with a beam energy of 18 MV using fractions of 1.8 Gy five times weekly up to 68.4 – 72 Gy (38–40 fractions) at the reference point (ICRU-50,16). An isocentric 4-field box technique consisting of anterior, posterior and two lateral fields (0°, 180°, 90° and 270°) was used in all cases.

All conformal 3D-plans were conventionally simulated before treatment. Simulator radiographs had been obtained in orthogonal (0°, 90°) projections and served as reference images for the position of bony landmarks and internal markers.

**Figure 1:**
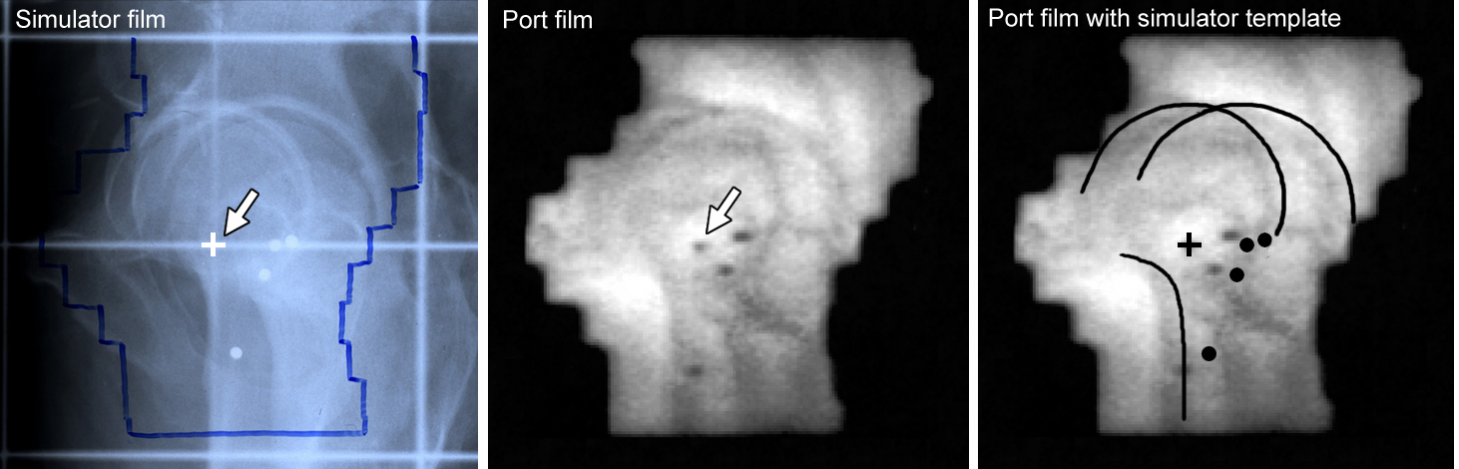
**Reference image (simulator film, left) and online image (port film, in the mid) are registered by the method shown on the right image**. A template containing isocenter, bony contours and radio-opaque markers is traced from the simulator radiograph and positioned on the portal image with isocenters and main axes in coincidence. The isocenter is shifted until the bony contours (setup error) or the implanted markers are in agreement (total error). For the motion error we determine the shift from the setup corrected position to the marker corrected position. The correction method is two-dimensional and performed separately for each projection (0° and 90°). Redundant measurements (in SI direction) are in good correlation (see text).

On each treatment day, patients were positioned using laser alignment to reference marks (one anterior and two lateral set-up crosses) on their skin. For all patients, weekly pre-treatment position verification with an EPID system (Siemens Beamview Plus™, Erlangen, Germany) was applied [17]. The electronic portal images (EPIs) for verification were acquired using 6 MV photons for the AP (0°) and left lateral (90°) fields once a week with 6 – 8 monitor units (MU), each before starting irradiation. The images were digitally processed (employing high frequency filters) to facilitate recognition of bony structures and radiopaque markers. On EPIs, the isocenter has been made visible by the projection of an isocenter marker (a 1.5 × 3 mm gold pin) located on the reticule. Bony landmarks and implanted markers were clearly identified on almost all portal films (Figure 1).

For the applied 2D/3D registration method, isocenter, bony contours and fiducial markers were drawn from the simulator films on transparent templates for every patient before irradiation. These templates were then used to match the reference images (0°, 90°) to the corresponding verification images manually.

**Figure 2:**
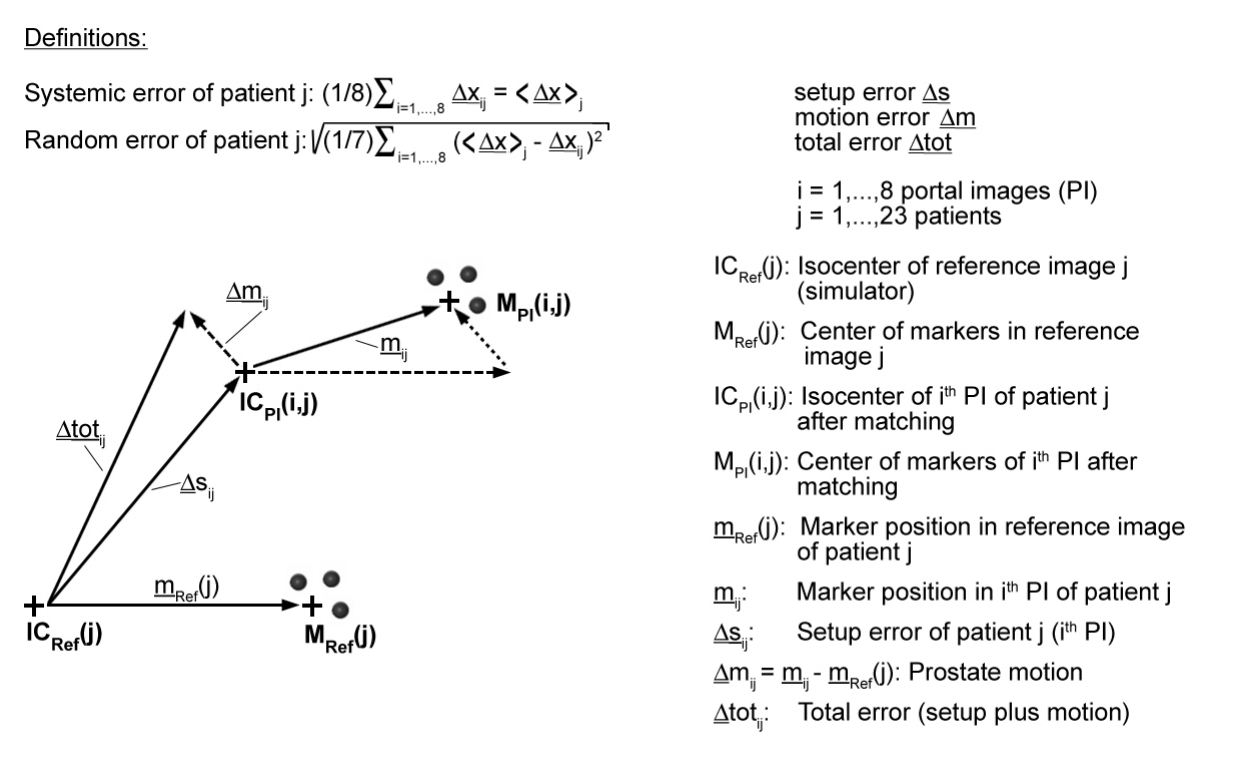
**Basic definitions of the different error components**: The setup error Δs and the motion error Δm can be added to the total error Δtot. For every patient j = 1...23 and portal image i = 1...8 the setup errors Δs_ij _are determined by matching the bony contours of the portal images (i, j) to the reference images j (simulator radiographs) according Figure 1. The motion errors Δm_ij _are determined after these matching procedures by subtracting the marker positions of the matched portal images (i, j) and the reference images j. The systematic error for a patient j is defined as the mean of single errors with respect to i = 1...8 portal images. The random error of patient j is defined as the standard deviation of this series. Then further statistical evaluation is possible, e.g. the mean systematic error for a series of patients (here 23 patients) in Table 2 and the mean standard deviations (mean random error) in Table 3.

An identical scale of the printed portal images and the templates was applied to determine the setup errors from the shift **Δs **of the isocenter (see Figure 2 for definition of symbols). The components of the vector **Δs **according to the main axes are determined providing the shift in left-right (LR) direction (lateral x-axis), anterior-posterior (AP) direction (vertical y-axis) and superior-inferior (SI) direction (longitudinal z-axis).

For evaluation and quantification of uncertainties, two orthogonal sets of 2D projections were available, firstly as reference images simulator radiographs and, secondly, the corresponding portal images. The AP beam provided data to detect the position of the landmarks and markers in the LR and SI direction and the lateral beam for the AP direction and SI direction as well. To identify the position of the target **m**, we used the arithmetic mean of the marker coordinates according to the isocenter (Fig. 2). All measurements were performed by the same author (RG). The consistency of the obtained deviations was tested by correlation of the corresponding values in SI direction taken from 0° and 90° projection. The correlation coefficient of r = 0.86 was satisfactory. The registration procedure takes about 3 minutes cumulating to a total treatment time of 6–8 minutes on average.

The evaluation procedure and the nomenclature are summarized in Figure 2. Firstly, we determined the vectorial displacements of the isocenters relative to the bony anatomy of the reference images **Δ**s_ij _for j = 1...23 patients and i = 1...8 weekly portal images per patient during the radiotherapy course yielding 8 × 23 = 184 setup errors (underlining identifying a vector). Secondly, the differences of the marker positions relative to the isocenters result in the prostate motion **Δ**m_ij_. Finally, the total displacement (setup error plus organ motion) of the target relative to the isocenter is calculated by Δtot_ij _= Δs_ij _+ Δm_ij_.

For all 184 fractions, mean and standard deviations for all kinds of errors (setup, motion, total) were calculated. We analysed the error distributions averaging over all fractions and patients.

Then, we determined means and standard deviations from 8 control EPIs for each patient resulting in the same error types **Δ**s(j), **Δ**m(j) and **Δ**tot(j) for j = 1...23 patients, and analysed the error distributions with respect to the patients. The standard deviations identify the *systematic errors *Σ(j) for every patient.

*Random errors ***σ**(j) for every patient j were calculated as standard deviations of the differences Δs(j) - Δs_ij _or Δm(j) - Δm_ij _or Δtot(j) - Δtot_ij _averaging over i = 1...8 PIs. We can also determine the mean random error for the entire group of patients averaging σ(j) over all j = 1...23 patients.

For correction of translational errors before treatment, we used an action level of 5 mm, i.e. all errors of 5 and more mm were corrected. The correction was performed on-line by repositioning the target according to the internal markers, moving the treatment couch manually. To calculate the minimum required margin width around the clinical target volume (CTV + margin = PTV), we utilized the prescription suggested by van Herk [18]. The margin around the clinical target volume (CTV) should be the sum of 2.5 times the standard deviation of the systematic total error (Σ) and 0.7 times the standard deviation of the random error (σ) to ensure a minimum dose of 95% to the clinical target volume for 90% of the fractions, i.e. allowing significant dose discrepancies in = 10% of sessions. If a position correction was performed (above the action level), we assume a residual error of = 3 mm [19] in all directions for the corrected fraction.

Statistical analysis was performed using JMP v7.0 (SAS Institute, Cary, NC, USA). Tests for sub-groups were performed using the paired t-Test.

## Results

We performed the analysis for 23 patients with 8 pairs of EPIs per patient, summing up to a total of 368 anterior-posterior and lateral port films in184 fractions. Bony contours, implanted markers and isocenter marker were clearly visible and evaluable in 96% of cases. All portal images were evaluable with respect to prostate motion employing the radiopaque markers. We had to replace only 1.8% of portal images due to insufficient identification of the bony structures.

**Figure 3:**
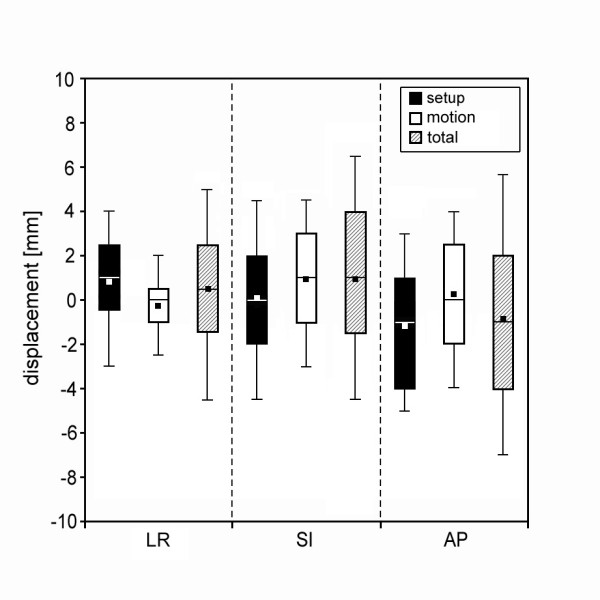
**Measured deviations in LR, SI and AP directions in a box plot format, showing the mean values (black squares), the median values (lines in the box) and the 10% (lower horizontal line), 25% (bottom of box), 75% (top of box) and 90% (upper horizontal line) percentile split in setup variability, prostate position variability and total error**.

As summarised in Table 1 we analysed all 184 fractions together and determined the displacements of the isocenter relative the bony anatomy (setup error), the displacement of the markers relative to the bony structures (prostate motion) and the displacement of the isocenter relative to the markers (combined or total targeting error). Figure 3 shows the measured deviations in a box plot format, indicating mean values, median values and selected percentiles from 10 to 90% (10%, 25%, 75%, 90%) in LR, SI and AP directions. The observed errors were greatest in the AP direction, where a range of 13 mm is found for the total deviation of the target (-7 to +6 mm) for 80% of the controls. The extremes observed in internal target motion were 8 mm in AP and 7 mm in SI direction.

**Table 1:** Setup error, motion error and total error

	Mean ± SD [mm] i = 1,..., 8; j = 1,..., 23	Range [mm]
**Setup error **Δs_ij_		
Left-right	0.8 ± 2.8	-8/10
Superior-inferior	0.1 ± 3.4	-9/14
Anterior-posterior	-1.2 ± 3.6	-15/9

**Prostate movement **Δm_ij_		
Left-right	-0.3 ± 1.8	-6/9
Superior-inferior	0.9 ± 2.8	-9/8
Anterior-posterior	0.3 ± 3.5	-10/10

**Total error **Δtot_ij_		
Left-right	0.5 ± 3.5	-10/19
Superior-inferior	0.9 ± 4.4	-14/13
Anterior-posterior	-0.8 ± 4.9	-19/14

Measured deviations for all 184 (= 23 × 8) portal imaging controls (averaged over i = 1,..., 8 weekly portal images in j = 1,..., 23 patients). Mean values <...> in all three directions (left-right LR, superior-inferior SI, anterior-posterior AP) ± standard deviations SD and ranges, i.e. extremal deviations in ± orientations of the axes, in LR, SI and AP direction for setup error <Δs_ij_>, prostate movement <Δm_ij_> and total errror <Δtot_ij_> are listed (see Fig. 2 for explanation of symbols).

We calculated the various errors for every patient separately (averaging over eight controls) and analysed the error distribution for 23 patients (see Table 2). Both, systematic setup and motion errors are in the range of ± 2 mm (± 1 SD) summing up to a total error of ± 3 mm.

**Table 2:** Systematic errors

	Mean ± SD [mm] j = 1,..., 23	Range [mm] j = 1,..., 23
**Systematic setup error **<Δs>_j_		
Left-right	0.8 ± 1.6	-2.9/3.9
Superior-inferior	0.1 ± 2.1	-2.7/6.2
Anterior-posterior	-1.2 ± 2.4	-5/4.1

**Systematic prostate movement **<Δm>_j_		
Left-right	-0.3 ± 1.1	-2.9/2.7
Superior-inferior	0.9 ± 1.9	-3.1/4.7
Anterior-posterior	0.3 ± 2.3	-4.3/5.0

**Systematic total error **<Δtot>_j_		
Left-right	0.5 ± 2.0	-2.6/6.6
Superior-inferior	0.9 ± 2.7	-4.3/7.3
Anterior-posterior	-0.8 ± 2.6	-6.7/4.0

Analysis of the mean vectorial setup errors per patient (after averaging over i = 1,..., 8 weekly controls) (1/8)Σ_i = 1...8 _Δs_ij _=: <Δs>_j_, the mean vectorial prostate movements (1/8)Σ_i = 1...8 _Δm_ij _=: <Δm>_j _and the mean vectorial total errors (1/8)Σ_i = 1...8 _Δtot_ij _=: <Δtot>_j_. After statistical analysis with respect to j = 1 to 23 patients we achieve the population based (mean) vectorial systematic error defined as (1/23)Σ_j = 1...23_<Δs>_j_, (1/23)Σ_j = 1...23_<Δm>_j _and (1/23)Σ_j = 1...23_<Δtot>_j _splitted in the RL, SI and AP direction and the standard deviation SD from these errors (see Fig. 2 for explanation of symbols). Also the range (minimum to maximum) is listed (right row).

For all 8 fractions per patient, the scatter (SD) about the individual averages (systematic errors) has been calculated providing the random errors for the different error types (Table 3). As expected the random errors are larger than the systematic errors and finally amount to total random errors of ± 4 mm in SI and AP direction, but only ± 3 mm in LR direction. The extremes can approach 1–1.5 cm, but these are rare cases.

**Table 3:** Random errors

	Mean [mm] j = 1...23	Range [mm] j = 1...23
**Random setup error**		
Left-right	2.3	-7.9/7.4
Superior-inferior	2.7	-8.6/7.8
Anterior-posterior	2.7	-12.1/5.4

**Random prostate movement**		
Left-right	1.4	-5.5/6.3
Superior-inferior	2.3	-5.9/8.6
Anterior-posterior	2.7	-7.0/8.0

**Random total error**		
Left-right	2.9	-8.0/6.6
Superior-inferior	3.9	-11.5/8.2
Anterior-posterior	4.3	-14.9/11.2

The standard deviations of measured deviations Δs_ij_, Δm_ij_, Δtot_ij _around the systematic errors (of each patient j = 1...23) <Δs>_j_, <Δm>_j_, <Δtot>_j _after averaging over i = 1,..., 8 weekly controls provide the random errors sqrt(1/7Σ_i = 1..8_(<Δs>_j _- Δs_ij_)^2^), sqrt(1/7Σ_i = 1..8_(<Δm_i_>_j _- Δm_ij_)^2^), sqrt(1/7Σ_i = 1..8_(<Δtot_i_>_j _- Δtot_ij_)^2^) for patient j = 1,..., 23. They are splitted into the RL, SI and AP direction for every error type, i.e. setup error, prostate movement and total error (see Fig. 1 for explanation of symbols). The mean random errors after averaging over 23 patients and their range, i.e. maximum deviations in both orientations of the axes, are given in the table.

The online protocol was applied at least 8 times per patient and, on average, 56% of the controls determined displacements of = 5 mm in at least one direction and had to be corrected. Margin calculations have been performed for each of the axes according to the prescription of van Herk [18] as described in section 2 (see Table 4). To consider for the total targeting error, the margins added to the CTV must be as large as 7.0 mm (LR) and 9.5 mm (SI, AP).

**Table 4:** Estimation of margins.

	**Random σ and systematic Σ error **[mm]						**Margin **[mm]		
**Direction**	LR		SI		AP		LR	SI	AP
	Σ	σ	Σ	σ	Σ	σ			
**No **correction	2.0	2.9	2.7	3.9	2.6	4.3	7.0	9.5	9.5
Correction **1×/week**	1.9	2.8	2.3	3.5	2.4	3.9	6.7	8.2	8.7
Correction **3×/week**	1.6	2.5	1.8	3.0	1.7	3.3	5.8	6.6	7.7
Correction **5×/week**	1.4	2.0	1.4	2.3	1.3	2.2	4.9	5.1	4.8

For our patient group the standard deviations of systematic errors (Σ) and random errors (σ) are listed in LR, SI and AP directions. Then the margins from CTV to PTV are calculated in all three directions according to the prescription 2.5 × Σ + 0.7 × σ [14] in order to consider these errors.

The errors are summarized in the first line as derived from the weekly portal images without any correction. In the following lines the variation is reduced by controling and correcting the position with increasing frequency per week (once, three times, every day). Correction is conducted if the threshold of 5 mm is crossed in any direction. After correction we assume a residual error of 3 mm in the respective direction. Under these circumstances the margins can be diminished from approximately 10 mm to 5 mm.

After position corrections once a week, these calculated margins reduce to 6.7, 8.2 and 8.7 mm. Therefore, a margin of 1 cm around the CTV is sufficient to counterbalance the set-up and internal motion inaccuracies if a weekly portal imaging with online correction is presumed. Gradual reduction of the errors and derived margins down to a minimum of 5 mm is obtained if the frequency of online control is further increased up to a daily correction as summarized in Table 4.

## Discussion

Various techniques have been developed to locate the prostate position on-line such as implanted fiducials (detected by X-rays), transabdominal ultrasound [20], electromagnetic tracking [21] and several kinds of in-room CT (e.g. [22]), in particular in conjunction with helical tomotherapy [23]. However, the highest precision is achieved by using intraprostatic markers.

The clinical use of implanted gold markers was found to be feasible in our hands. The geometrical center of implanted radiopaque markers characterizes the prostate position. Several groups have investigated the possibility of seeds migration and have found no or only little motion [24,25]. In addition, the reliability of markers for the location of the prostate has been questioned because of interfraction rotation or deformation [26], but these factors leave the prostate dosimetry unaffected [27]. The analysis is standardized so that the interobserver variability is low. Therefore implanted markers and EPID based methods are used for targeting in radiotherapy of prostate cancer with increasing frequency.

Our results provide information about the scatter of target positions during radiotherapy. Setup inaccuracies were reviewed by Hurkmans [28]. In his analyses data were obtained from repeated simulations, from EPID studies and from repeated CT scans. The standard deviations of the setup errors ranged from 1 to 4 mm, which is in accordance with our results. We also found standard deviations below 4 mm. Analysis of the contributions to the total targeting error indicates, that the setup errors cause approximately one half of the entire target position variability and offers a potential improvement in total target positioning.

The prostate position can move relative to the skeleton [4]. An overview of interfraction prostate motion studies was presented in a paper by Langen [29]. The position of the prostate at the time of treatment can be visualized with a variety of techniques, and differences in measurement techniques make it difficult to compare the results of published studies. In summary, the SDs of the prostate motion range in the LR direction from 0.7 to 1.9 mm, in SI from 1.7 to 3.6 and for AP from 1.5 to 4.0 mm. We measured for prostate motion in RL, SI and AP standard deviations of 1.8, 2.8 and 3.5 mm, even though some extremes of motion were registered in a few patients (table 1). Thus, our results are in general agreement with literature [30-34].

We found the largest errors, for both, setup as well as prostate motion, in the AP direction, followed by SI and LR directions in accordance with the series of Beaulieu and others [14,29,35]. Along the lateral axis the prostate is confined within the pelvis and published data show only small deviations in this direction. In our study, the distribution of organ motion and setup errors for translation is in the range of the published values [36], e.g. 90% of the observed displacements were 7 mm or less.

Interfraction position variation of the prostate as a source of treatment error is mainly caused by variable fillings of the bladder and/or rectum that displace the prostate mainly in SI and AP direction as shown by magnetic resonance imaging of the pelvis [27]. Patient instructions attempt to prepare rectal and bladder distension in a standardized way before treatment. This may reduce the frequency of large prostate movements, but does not eliminate the motion error [21]. There is even an intrafractional motion of 1–3 mm on average [37] and after initial positioning the displacement of the prostate gland increases with elapsed time. This matter raises concerns with regard to correction for misalignments [38] and the treatment time of 20–30 minutes per session using novel techniques i.e. intensity modulated radiotherapy, tomotherapy etc., which will induce a new intrafraction errors. Recently published analyses of this issue indicate that a 3-mm planning target margin is in most cases sufficient to account for intrafractional motion [39].

Both uncertainties, setup error and target motion can be split into random and systematic deviations. The systematic component of setup error is largely caused by the systematic error inherent to the use of a reference image obtained by use of the planning-CT. The random component of the setup error is mainly caused by uncertainties from utilisation of skin markers, while the random error of target position is mainly caused by organ movement, respectively. We found for setup, prostate location variation and combined error in general larger random errors than systemic errors, obviously due to the reduction of systematic errors by the weekly performed corrections.

In our study we calculated necessary CTV-PTV margins (without correction) of 7.0 to 9.5 mm (RL, SI and AP direction) according Table 4. Similar margins (without correction) are reported by Kupelian [38] with 10, 10 and 12 mm, McNair [40] with 5, 7.5 and 11 mm and van den Heuvel [41] with 9.5, 8.6 and 10 mm.

According to the formula given in Section 2 to estimate the margin between CTV and PTV [18], systematic errors have the largest impact on the size of PTV margins. Therefore, offline correction protocols attempt to determine and correct the systematic error. They have the advantage to be effective despite a low imaging frequency. Different offline protocols have been successful implemented into clinical practice [42,43]. On the other hand, Litzenberg [44] figured out, that because of changes in patient's setup characteristics off-line protocols, especially those directed to localize the prostate using markers did not show any significant benefit in reducing the total error of implanted fiducial gold markers in 10 prostate cancer patients in comparison to daily online position correction. For the same reasons, applying these methods directly to the implanted markers also gave larger residual errors than expected. It may be difficult to identify patients who would benefit from off-line protocols and those who may require daily on-line corrections [44].

Evaluating their possible benefit, on-line correction protocols have the potential to reduce both systematic and random errors, but at the expense of increasing treatment time per fraction. As expected, systematic errors are effectively reduced with increasing imaging frequency [38]. After one weekly online correction and 5 mm action level, we found margins of about 7 to 9 mm. These margins can be further reduced to a minimum of 5 mm by increasing the control frequency (Table 4). Kupelian [38] calculated treatment margins for 8 different potential non-daily imaging strategies, among them low-workload weekly protocols. For a weekly online protocol with 3-mm threshold, he found margins of 8, 8 and 6 mm (LR, SI and AP), which agrees quite well with our results.

A daily positioning correction is feasible under routine conditions employing the new generation of linear accelerators with image guidance (on-board imaging or x-ray tracking). Using these techniques the residual error can be further decreased below 3 mm and the required safety margin is reduced down to 3 mm (unpublished data). An accuracy of only 5 mm is achieved using megavoltage CT without intraprostatic markers [38].

## Conclusion

In summary, correction of setup errors alone is not sufficient because target motion contributes significantly to positioning inaccuracies. The implantation of gold markers for a correction protocol was feasible in our study. A weekly on-line setup verification employing these radiopaque markers and megavoltage radiography results in CTV-PTV margins of 7 to 8.5 mm. More effort can furthermore decrease these margins. A correction of three times per week leads to margins of 6 to 7.5 mm, and daily corrections can further reduce the margin down to 5 mm.

## Competing interests

The authors declare that they have no competing interests.

## Authors' contributions

RG analysed the simulator films and portal images, determined the errors, performed the statistical analysis and drafted the manuscript. PW initiated the study, treated the patients, formulated the mathematical background and revised the first draft of the manuscript. VB participated in designing the study and approved the treatment concepts. DB coordinated the recruitment of patients and data acquisition. All authors participated in the critical discussion of the data and their statistical analysis. All authors improved the manuscript and approved the final version.
